# The venetian-blind effect: a preference for zero disparity or zero slant?

**DOI:** 10.3389/fpsyg.2013.00836

**Published:** 2013-11-11

**Authors:** Björn N. S. Vlaskamp, Phillip Guan, Martin S. Banks

**Affiliations:** ^1^Vision Science Program, School of Optometry, University of BerkeleyBerkeley, CA, USA; ^2^Philips ResearchEindhoven, Netherlands; ^3^The UC Berkeley UCSF Graduate Program in Bioengineering, University of California, BerkeleySan Francisco, CA, USA

**Keywords:** venetian blinds, wallpaper illusion, cross-correlation, disparity, slant

## Abstract

When periodic stimuli such as vertical sinewave gratings are presented to the two eyes, the initial stage of disparity estimation yields multiple solutions at multiple depths. The solutions are all frontoparallel when the sinewaves have the same spatial frequency; they are all slanted when the sinewaves have quite different frequencies. Despite multiple solutions, humans perceive only one depth in each visual direction: a single frontoparallel plane when the frequencies are the same and a series of small slanted planes—Venetian blinds—when the frequencies are quite different. These percepts are consistent with a preference for solutions that minimize absolute disparity or overall slant. The preference for minimum disparity and minimum slant are identical for gaze at zero eccentricity; we dissociated the predictions of the two by measuring the occurrence of Venetian blinds when the stimuli were viewed in eccentric gaze. The results were generally quite consistent with a zero-disparity preference (Experiment 1), but we also observed a shift toward a zero-slant preference when the edges of the stimulus had zero slant (Experiment 2). These observations provide useful insights into how the visual system constructs depth percepts from a multitude of possible depths.

## Introduction

The perception of depth from binocular disparity depends on correctly matching corresponding features in the left- and right-eyes' images. Complex scenes present multiple candidate features for matching so the visual system must prune false matches in order to attain a correct interpretation of the 3D scene. The prevailing model used to determine correspondence is based on correlation. Estimating disparity by local cross-correlation of the two input images has been used successfully in computer vision (Kanade and Okutomi, 1994; Clerc and Mallat, 2002) and in modeling human vision (Cormack et al., 1991; Fleet et al., 1996; Harris et al., 1997; Banks et al., 2004; Filippini and Banks, 2009). Local cross-correlation is an appropriate computational analog for the disparity-energy units that underlie binocular interaction in visual cortex (Ohzawa et al., 1990; Prince and Eagle, 2000; Cumming and DeAngelis, 2001). However, as we will see, there must be significant computations beyond correlation to explain various stereo phenomena.

### Wallpaper illusion

**Figure 1:**
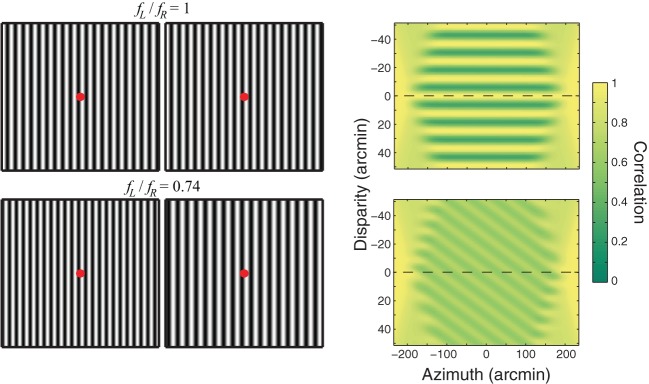
**Wallpaper illusion and Venetian-blind effect.** Left: The upper part shows the Wallpaper illusion. Vertical sinewave gratings of the same spatial frequency and contrast are presented to the two eyes (ratio of spatial frequencies *f*_*L*_*/f*_*R*_ = 1). Cross-fuse such that the red dots superimpose. One sees a single plane of vertical stripes at the depth of the fused red dot. The lower part shows the Venetian-blind effect. Vertical sinewave gratings of different spatial frequencies are presented to the two eyes (*f*_*L*_*/f*_*R*_ = 0.74). Cross-fuse again such that the red dots superimpose. Holding fixation on the fused red dot, one sees a series of slanted planes: Venetian blinds. If you move your eyes slowly to the left or right, the breaks in the blinds move with the eyes. Right: Local cross-correlation to the stimuli on the left. In each case, horizontal position is plotted on the abscissa and disparity on the ordinate. The left- and right-eye images were sampled with an isotropic Gaussian window with a standard deviation of 20 arcmin. Correlation is represented by intensity. The dashed horizontal lines represent zero disparity when the eyes are fixating such that the red dots superimpose. For the Wallpaper stimulus, a series of ridges of high correlation are observed. Their separation is equal to the period of the sinewave grating. For the Venetian-blind stimulus, a series of ridges of high correlation is observed again, each ridge rotated from frontoparallel. The peak correlations are lower than with the Wallpaper stimulus.

Periodic stimuli like the ones in Figure 1 present an interesting challenge to models of stereo correspondence. When identical periodic stimuli are presented to the two eyes (upper row), the *Wallpaper illusion* occurs. One sees a single plane and the apparent depth of the plane is closely associated with the distance to which the eyes are converged (Brewster, 1844; McKee et al., 2007). That is, if the eyes converge, the plane is seen as near and if the eyes diverge, the plane is seen as far. The phenomenon is called an illusion because the stimulus provides multiple correlation solutions but only one is seen. The upper right panel of Figure 1 shows that cross-correlation of the images yields a multitude of correlation peaks. The same behavior is observed in disparity-selective V1 neurons; when presented periodic stimuli, these neurons exhibit multiple response peaks (Cumming and Parker, 2000). Therefore, if the disparity estimates obtained by local cross-correlation or the responses of individual V1 neurons were the sole determinant of perceived depth, the wallpaper stimulus should look like multiple parallel planes stacked in depth rather than a single plane. The fact that the stimulus does not appear that way means that processes beyond correlation are involved in constructing the final percept.

### Venetian-blind effect

When the spatial frequencies of periodic stimuli differ significantly in the two eyes, as shown in the lower row of Figure 1, the *Venetian-blind effect* occurs. One sees a series of small slanted planes (the Venetian blinds; Cibis and Haber, 1951; Tyler and Sutter, 1979; Halpern et al., 1996). Cross-correlating the images again yields a multitude of correlation peaks (lower right panel). If the disparity estimates obtained by correlation were the sole determinant of perceived depth, the Venetian-blind stimulus should appear to have multiple slanted planes stacked in depth. Again this is not what one perceives.

### Minimum absolute disparity and minimum relative disparity rules for correspondence

Various computational strategies have been proposed for eliminating false matches and assuring stable depth percepts. Central to most of these strategies is the idea that some disparity patterns should be preferred *a priori* to others.

Two common strategies for stereo correspondence are the minimum absolute disparity and minimum relative disparity rules. These rules have been referred to as the nearest-neighbor and nearest-disparity rules, respectively. To explain these rules, we must first define absolute and relative disparity. Suppose that the eyes are fixating a point *Q* in space: i.e., the image of *Q* falls on the foveas. Another point *P* at a different distance creates images at different positions on the left and right retinas. α_*L*_ is the angle between *Q* and *P* seen by the left eye and α_*R*_ is the corresponding angle seen by the right eye. The absolute disparity of *P* is the angular difference α_*R*_–α_*L*_. Thus, absolute disparity uses the fixated point (in this case, *Q*) as the reference for the disparity of another point (*P*). If the eyes fixate some other point, the relative disparity between *Q* and *P* remains α_*R*_–α_*L*_. Thus, relative disparity is a measure between two points whether they are fixated or not.

The *minimum absolute disparity rule* attempts to minimize the absolute disparity of image features. That is, the rule selects correspondence solutions that place the image features closest to the horopter (Arditi et al., 1981). This rule can be implemented by adding a weighting function, centered at zero disparity, to the local cross-correlation model. One can implement this by, for example, multiplying the correlation output by a Gaussian ridge centered at zero. As a result, solutions near the fixation distance are favored over solutions in front of and behind that distance.

The *minimum relative disparity rule* attempts to minimize the relative disparity of image features. That is, the rule selects correspondence solutions that minimize the difference in disparity between nearby points (Marr and Poggio, 1976; Mitchison and McKee, 1987). The minimum relative disparity rule can be described as a “smoothness” constraint and has been implemented through application of coarse-to-fine matching (Marr and Poggio, 1979) and spatially weighted smoothing (Qian and Zhu, 1997). The local cross-correlation model yields smooth solutions because it uses relatively large spatial samples as input to the correlation stage (Kanade and Okutomi, 1994; Banks et al., 2004).

In the Venetian-blind stimulus, the minimum relative disparity rule would favor a solution of a single slanted plane. Instead the percept is a series of small planes each centered in the fixation plane. The perceived solution minimizes absolute disparity (because the perceived jagged surface is close to the horopter) and is therefore consistent with the minimum absolute disparity rule.

Applying a weighting function centered on zero disparity to the output of the correlation stage can qualitatively account for the solutions obtained in the Wallpaper and Venetian-blind effects. In forward gaze, however, a zero-disparity weighting function is identical to a zero-slant function, so we cannot determine whether such a weighting function or preference is based on disparity or on slant. There are good reasons for both and we examine those reasons next.

### Evidence for zero-disparity preference

Most binocular neurons have a preference for a disparity near zero. For example, Cumming and Parker (1999) measured disparity-tuning curves for 53 V1 neurons in the parafovea and near periphery of primates. The average preferred disparity was −0.07 arcmin and the standard deviation was 3.67 arcmin. Thus, the preferred disparities clustered around 0. Because of this propensity, many models of disparity processing in visual cortex build in a bias for zero disparity (Fleet et al., 1996; Qian and Zhu, 1997). Points along the geometric horopter (i.e., the Vieth-Müller Circle) have, by definition, a disparity of zero, so a zero-disparity preference would be a bias toward the horopter. Depth estimates from disparity are most precise for stimuli on or near the horopter (Blakemore, 1970; Held et al., 2012) presumably because the brain devotes more resources for disparity processing of points near the horopter. For these reasons, it makes sense that there would be a preference for correspondence solutions that minimize absolute disparity.

### Evidence for a zero-slant preference

There is a geometric basis for a zero-slant preference. Consider an observer viewing a world of small surfaces that vary randomly in 3D orientation. Retinal images created by such surfaces project to an area that is proportional to the cosine of the slant; that is, retinal-image area is on average maximized when the slant is zero and goes to zero when the slant is 90°. This statistical regularity could reasonably be incorporated into visual processing as a prior for zero slant. A variety of perceptual phenomena are consistent with such a prior (Perrone, 1982).

**Figure 2:**
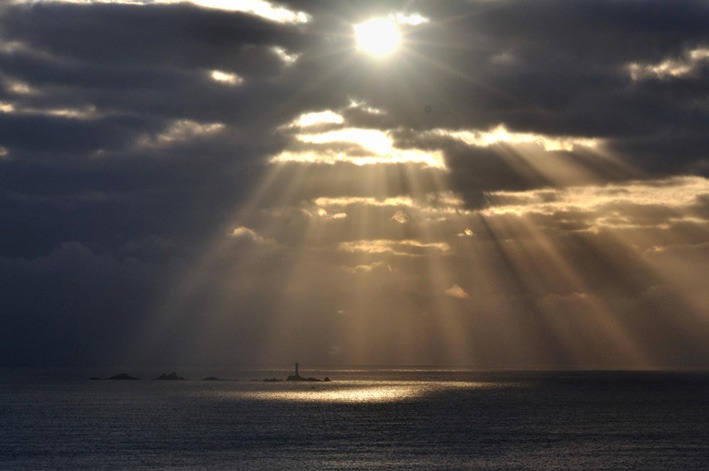
**The sunbeam illusion or crepuscular rays.** The sun shines through clouds creating regions of illumination and shadow. The beams appear to radiate from a central point much as the blades of a fan emanate from a center. The perceptual effect is striking in the natural environment when the viewer looks with one eye while not moving the head. Image: Andrew Green “over the lighthouse,” December 13, 2010 via Flickr, Creative Commons Attribution.

The photograph in Figure 2 shows rays of sunlight shining through clouds. The rays appear to radiate like a fan from a central point; the fan appears to lie in a plane perpendicular to the viewer's line of sight. The same effect occurs in the natural environment when viewed with one eye and a stationary head. Despite the appearance of a fan-like pattern, the rays are actually all parallel to the line of sight because their source is the sun, which is effectively at infinite distance. Nonetheless, the rays regress perceptually toward a fronto-parallel plane, so they appear to lie in a surface of zero slant even though their true geometry is parallel rays with slants close to 90°. This effect has been called the sunbeam illusion or crepuscular rays (Minnaert, 1954; Lynch, 1987).

There is yet another reason for hypothesizing that the Venetian-blind effect manifests a bias toward zero slant. In the natural environment, the slant of a surface is specified by a variety of cues including disparity and perspective (e.g., the texture gradient and relative size). In estimating the slant of a surface, the visual system integrates the information provided by disparity and perspective in a statistically optimal fashion (Knill and Saunders, 2003; Hillis et al., 2004). The Venetian-blind stimulus presents conflicting disparity and perspective cues to slant. When the ratio of spatial frequencies presented to the two eyes differs from 1, disparity specifies a slanted plane. But the density of texture never differs from one stimulus region to another, which specifies a slant of zero (Wilson, 1976). Thus, when the frequency ratio becomes large and disparity information becomes difficult to compute, the percept may regress toward a slant of zero because that is consistent with the slant specified by perspective.

Another example is provided by the equidistance tendency (Roelofs and Zeeman, 1957; Gogel, 1965). When reliable information is available for the distances of two objects, observers see the stimuli at distances that are reasonably consistent with their actual distances. However, as distance information is removed (e.g., monocular viewing, no head motion), observers tend to see stimuli at the same distance; the stimuli have apparently regressed into a virtual surface with a slant of zero.

### Experimental question

**Figure 3:**
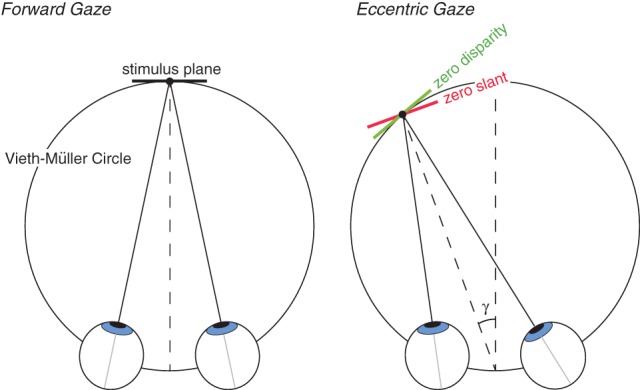
**Zero disparity and zero slant for different directions of gaze.** Left: The eyes are in forward gaze. The zero-disparity and zero-slant surfaces are both tangent to the Vieth-Müller Circle and are therefore identical. Right: The eyes are in leftward gaze. The zero-disparity surface is again tangent to the Vieth-Müller Circle, but the zero-slant surface is not. The slants of the two surfaces differ by γ, the horizontal version of the eyes. Thus, with viewing in eccentric gaze, the predictions for zero-disparity and zero-slant preferences differ.

We asked whether the pruning of false matches that occurs in stereo correspondence is more consistent with a preference for zero disparity or zero slant. Specifically, we presented periodic stimuli of different spatial frequencies to the two eyes and found the frequency ratios at which multiple planes (i.e., Venetian blinds) are perceived as opposed to a single plane. We dissociated the predictions of the zero-disparity and zero-slant preferences by presenting the stimuli in eccentric gaze where the zero-disparity and zero-slant surfaces differ by the eccentricity of the viewer's gaze direction (Figure 3).

## Experiment 1

### Methods

#### Participants

There were four participants (two males) all of whom had normal or corrected-to-normal vision and good stereo-acuity. One was author BV; the others were naive with respect to the experimental hypotheses. All were trained psychophysical observers.

#### Materials

We used a haploscope to present the binocular stimuli (Backus et al., 1999). It consisted of two CRTs (Viewsonic, h225f, 2048 × 1536 pixels, pixel size of ~1.6 arcmin, refresh rate of 75 Hz), one for each eye. The CRTs were placed on rigid arms that rotated about axes that were co-linear with the vertical rotation axes of the observer's eyes. The CRTs were 39 cm from the eyes and they were perpendicular to the lines of sight when the observer fixated the screen centers. To assure accurate positioning of the eyes relative to the apparatus, head position was adjusted and fixed with an adjustable bite bar (Backus et al., 1999; Hillis and Banks, 2001). With this setup, eye position could be varied without affecting the retinal images. Vergence distance was always 25 cm. The vergence angle varied slightly across observers because their inter-ocular distances varied; vergence angle also varied slightly with version. Stimuli were created and presented with Matlab and the Psychophysics Toolbox (Brainard, 1997; Pelli, 1997).

#### Stimuli and procedure

Stimuli were similar to those that give rise to the Wallpaper illusion (Figure 1). They consisted of vertical sinewave gratings with an average luminance of 33.4 cd/m^2^ and contrast of ~1. The stimulus was viewed through square 8.1 × 8.1° apertures 25 cm from the eyes. The images of the apertures were identical in the two eyes and therefore had a disparity of zero.

The average spatial frequency of the gratings presented to the two eyes was 0.5, 1, 2, or 4 cycles per degree (c/deg). Different frequencies were presented to the left and right eyes by changing frequency by equal and opposite amounts in the two eyes such that one eye received an image of higher spatial frequency than the other. We express the frequency difference as a ratio:

$$f_{ratio}=f_{L}/f_{R}$$

where *f*_*L*_ and *f*_*R*_ are the spatial frequencies in the left and right eyes, respectively. When the ratio is slightly greater than 1, a slanted plane is perceived with the right side farther than the left. When the ratio is much greater than 1, the plane appears to separate into a series of smaller slanted planes: this is the Venetian-blind effect. When the ratio is less than 1, the perceived slants of the overall plane and the Venetian blinds are opposite in direction. The breakup from a single slanted plane to the Venetian blinds can be seen in Supplementary Video 1.

A binocular fixation point was always present. Observers initiated each 1-s stimulus presentation with a key press. After the stimulus was extinguished, observers indicated whether they had seen blinds or not. The frequency ratio was adjusted trial by trial according to an adaptive procedure (Quest; Watson and Pelli, 1983). The procedure sought the ratio at which blinds were seen on 50% of the trials. In a session for a given experimental condition, six procedures of 25 trials each were run in interleaved fashion. Three were for frequency ratios less than 1 and three for ratios greater than 1. They were all constrained to never cross 1. The session was repeated later on so that each experimental condition contained a total of 300 trials. The resulting responses were accumulated and fitted with cumulative Gaussians using a maximum-likelihood criterion (Wichmann and Hill, 2001). For each condition, one Gaussian was fit to the data for ratios less than 1 and another Gaussian to the data for ratios greater than 1. The 50% points on those two functions were the estimates of the two ratios that yielded perceived blinds half of the time.

We presented stimuli at three different values of horizontal version: −16, 0, and +16°, which corresponded respectively to leftward, straight ahead, and rightward gaze. Each combination of version and average spatial frequency was presented a total of 300 trials.

### Results

**Figure 4:**
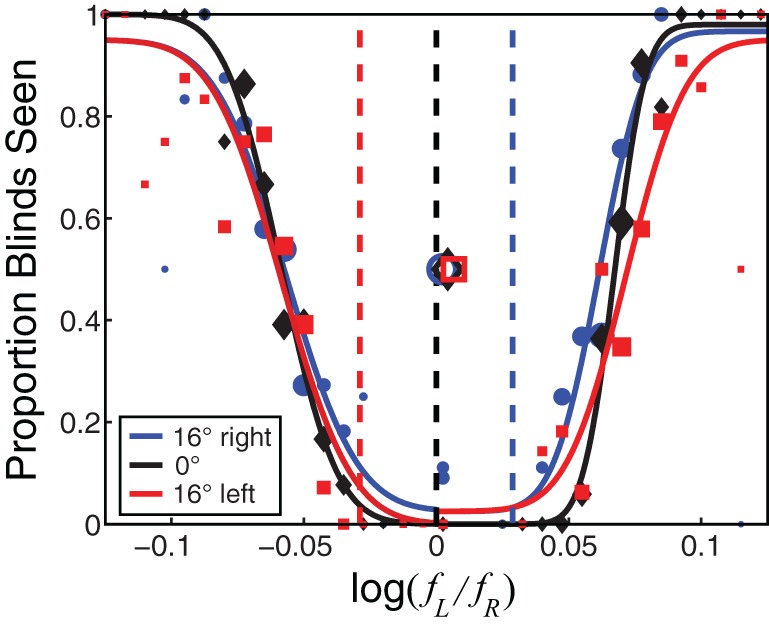
**The probability of seeing Venetian blinds as a function of inter-ocular frequency ratio.** The proportion of trials on which blinds were reported is plotted as a function of the logarithm of the inter-ocular frequency ratio. The red circles, black squares, and blue diamonds represent the data for leftward, forward, and rightward gaze, respectively. The size of the data symbols is proportional to the number of trials presented at that particular frequency ratio. The curves are cumulative Gaussians fit to the data. The open data symbols are the average of the two ratios that produced blinds on half the trials (i.e., the average of the 50% points on the two cumulative Gaussians fit to each data set.) The black dashed vertical line is the prediction for zero disparity for leftward, forward, and rightward gaze. The red, black, and blue dashed lines are the predictions for zero slant for leftward, forward, and rightward gaze, respectively.

Figure 4 plots the psychometric data for one observer at an average spatial frequency of 1 c/deg. The proportion of stimulus presentations on which blinds were reported is plotted as a function of the inter-ocular frequency ratio. Red, black, and blue symbols indicate the data for leftward, forward, and rightward gaze, respectively. When the frequency ratio was close to 1, the observer never saw blinds no matter what the gaze direction was. When the ratio was much less than or much greater than 1, the observer always saw blinds regardless of the gaze direction.

**Figure 5:**
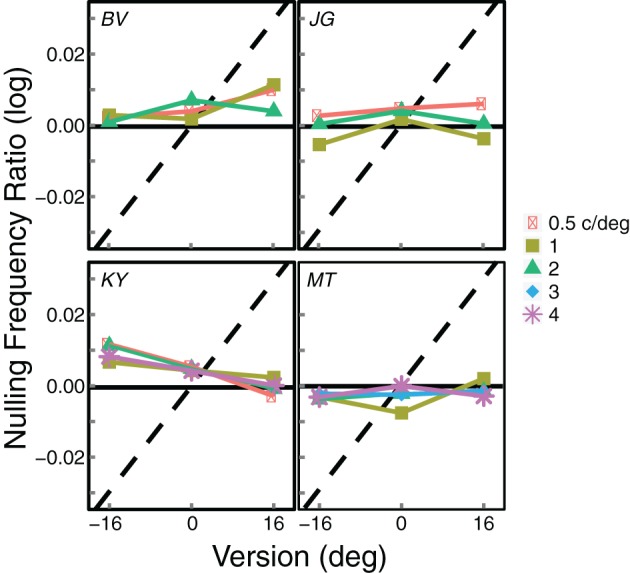
**Summary results from Experiment 1.** Each panel shows the results from an individual observer. The logarithm of the average of the inter-ocular frequency ratios that produced visible blinds (that is, the average of the 50% points on the two cumulative Gaussians fit to the psychometric data) is plotted as a function of gaze direction. Different symbols represent the data from different average spatial frequencies. The black horizontal lines represent the predicted values if the frequency ratio at which blinds were perceived is based on disparity. The dashed diagonal lines represent the predictions if blinds perception is based on slant; those predictions differ slightly across observers because of their differing inter-ocular distances.

Figure 5 summarizes all of the data, a different panel for each observer. The panels plot the logarithm of the average of the inter-ocular frequency ratios at which blinds were perceived: i.e., the average of the 50% points on the two cumulative Gaussians fit to the psychometric data. Gratings of 0.5 and 4 c/deg did not always give rise to Venetian blinds for all participants and are therefore not included in all panels. Observer MT ran an extra condition at 3 c/deg.

If a preference for zero disparity determines the appearance of blinds, they should appear at the same frequency ratio for all gaze directions. This prediction is represented by the horizontal solid lines. If a zero-slant preference determines blinds, they should appear at different frequency ratios for each gaze direction. This prediction is represented by the diagonal dashed line in each panel. The data are very consistent with the zero-disparity prediction. When the data deviate from this prediction, they do so in a direction opposite from the zero-slant prediction.

We calculated squared errors between the data and the two predictions for leftward and rightward gaze, all spatial frequencies, and all observers (we excluded the forward-gaze data because the predictions for that condition are identical). The average squared error between the data and zero-disparity predictions (in log frequency ratio) was 0.0002 (*SE* = 0.0001). The average squared error for zero slant was much greater at 0.0123 (*SE* = 0.0024). This shows again the data are much more consistent with the zero-disparity predictions than with the zero-slant predictions.

## Experiment 2

In the first experiment, the aperture was identical in the two eyes and therefore had zero disparity. We were concerned that such an aperture could bias the appearance of Venetian blinds toward zero disparity. To test this possibility, we redid the first experiment but with an aperture that had zero slant rather than zero disparity.

### Methods

The apertures were software-clipping windows creating an apparent aperture of zero slant for each gaze direction. The aperture subtended 8.1 × 8.1° in forward gaze in Experiment 1. The aperture subtended 8.3° horizontally in the left eye and 7.8° horizontally in the right eye when the eyes were in leftward gaze and 7.8° in the left eye and 8.3° in the right when the eyes were in rightward gaze. The stimuli within the aperture were the same as in Experiment 1.

### Results

**Figure 6:**
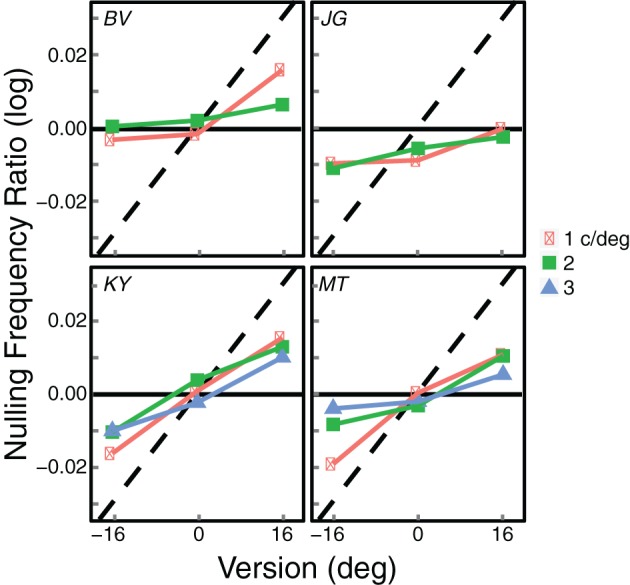
**Summary results from Experiment 2.** Each panel shows the results from an individual observer. The logarithm of the average of the inter-ocular frequency ratios that produced visible blinds is plotted as a function of gaze direction. Different symbols represent the data from different average spatial frequencies. The black horizontal lines represent the predicted frequency ratios for seeing blinds based on disparity. The dashed diagonal lines represent the predictions for blinds perception based on slant; those predictions differ slightly across observers because of their differing inter-ocular distances.

The summary data from this experiment are shown in Figure 6. The data were again most consistent with the zero-disparity prediction, but they deviated from the disparity prediction in the direction of the zero-slant prediction. This is most evident for observers MT and KY when the average frequency was 1 c/deg. In those two cases, the zero-slant prediction was slightly more accurate than the zero-disparity prediction. However, for all the other conditions and observers zero disparity was still the better predictor for seeing blinds. To quantify the correspondence between the data and the zero-disparity and zero-slant predictions, we again calculated squared errors between the data and predictions for all leftward and rightward gaze, all spatial frequencies, and all observers. The average squared error for the zero-disparity predictions was 0.0012 (*SE* = 0.0004) while the average error for the zero-slant predictions was considerably greater at 0.0039 (*SE* = 0.0003). To determine if this difference was statistically significant, we computed the ratio of the difference between the data and the zero-disparity predictions divided by the difference between the zero-disparity and zero-slant predictions. This ratio would be 0 if zero-disparity were a perfect predictor and 1 if zero-slant were a perfect predictor. A ratio of 0.5 would indicate that the two are equally good predictors. The average ratio was 0.215, which is significantly smaller than 0.5 [*t*_(3)_ = 3.255, *p* = 0.0473]. Thus, the data in Experiment 2 were significantly closer to the zero-disparity predictions than to the zero-slant predictions.

## Discussion

### Zero-slant aperture effect on the perception of venetian blinds

When we used an aperture consistent with zero slant (Experiment 2) rather than zero disparity (Experiment 1), the data shifted in the direction of the zero-slant predictions. Specifically, the frequency ratios at which blinds were observed changed slightly when the disparity of the aperture was changed. To see if this shift can be understood from correlating the two eyes' images, we applied local cross correlation to the stimuli when the aperture was zero disparity and when it was zero slant. The correlation outputs were nearly identical in the two cases, so there is nothing obvious in the inter-ocular correlation that would explain the shift in the data.

Mitchison and McKee (1987) presented ambiguous periodic stereograms and showed that the disparity of the edges biased the perceived depth of the rest of the stimulus. The change we observed between Experiments 1 and 2 is similar to this observation and points to a role of edges in determining binocular matches in the rest of the stimulus.

### Phenomenology of wallpaper and venetian-blind effects

Two aspects of the phenomenology of the Wallpaper and Venetian-blind effects are additionally informative about the process of stereo correspondence.

**Figure 7:**
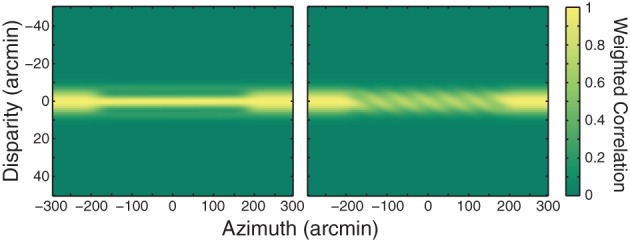
**Weighting function applied to cross-correlation output.** A Gaussian ridge with a standard deviation of 4.5 arcmin was multiplied by the correlation outputs from Figure 1. The ridge is centered on the fixation distance.

First, when the blinds are perceived, the perceived edges are generally not vertical. For example, when the inter-ocular frequency ratio is greater than one (higher spatial frequency in the left eye), the edges are tilted clockwise relative to vertical. When the ratio is less than one, the edges are tilted counter-clockwise. This apparent rotation of the edges of the blinds is probably caused by the pitch of the empirical vertical horopter (Nakayama, 1977; Hibbard and Bouzit, 2005; Cooper et al., 2011). The pitch occurs because positions of corresponding points above and below the foveas are sheared such that points in the upper visual field require uncrossed disparity to be stimulated while points in the lower field require crossed disparity. If the preference for zero disparity is tied to empirical corresponding points rather than geometric corresponding points, the weighting function illustrated in Figure 7 would be shifted to uncrossed disparities in the upper visual field and to crossed disparities in the lower field. One can see by reference to that figure that this would cause a break in the solution at more distant points above fixation and at nearer points below. This in turn would cause clockwise rotation of the edges when the slant is positive (frequency ratio greater than 1) and counter-clockwise rotation when the slant is negative (ratio less than 1). We believe therefore that the perceived orientation of the edges reveals that the preference for zero disparity is defined relative to empirically rather than geometrically corresponding points.

The second observation also concerns the appearance of the blinds. Careful inspection of the Venetian-blind stimulus in the lower part of Figure 1 reveals that multiple blinds are never seen in a given visual direction. The perceived depth is jagged, but there is only one perceived depth in each visual direction. We argued that the Wallpaper and Venetian-blind effects can be accounted for by applying a weighting function centered at zero disparity to the output of disparity-estimation processes. Although this goes a long way toward accounting for these effects, such a simple weighting process cannot explain why only one depth is perceived per direction. To explain this, one needs to invoke a higher-order constraint such as the suppression of additional solutions in a given direction. Such a higher-order constraint would eliminate or greatly reduce the probability of stereo transparency.

### Relative-disparity minimization and cross correlation

A smoothness constraint is often used in models of stereo correspondence. Such a constraint is meant to favor solutions that minimize the difference in disparity between nearby points (Marr and Poggio, 1976, 1979) and is consistent with the minimum relative disparity rule. The constraint makes sense for two reasons. First, most surfaces in the natural environment are opaque so only one surface can be seen in a given visual direction. Second, most scenes are locally continuous in depth. Of course, neither of these properties is always followed: opacity is violated by specular reflections (i.e., viewing the reflection off the surface of a dirty mirror) and transparency, and continuity is violated at the occluding boundary of closed objects. Nonetheless, the properties are generally followed, so the smoothness constraint is reasonable for natural scenes.

It is hard to see how the perception of Venetian blinds is consistent with the smoothness constraint. The smoothness constraint should yield the percept of a smooth slanted plane rather than the jagged blinds that are actually observed. We examine this paradox by considering ambiguous stimuli that often yield percepts consistent with minimum relative disparity rather than minimum absolute disparity.

**Figure 8:**
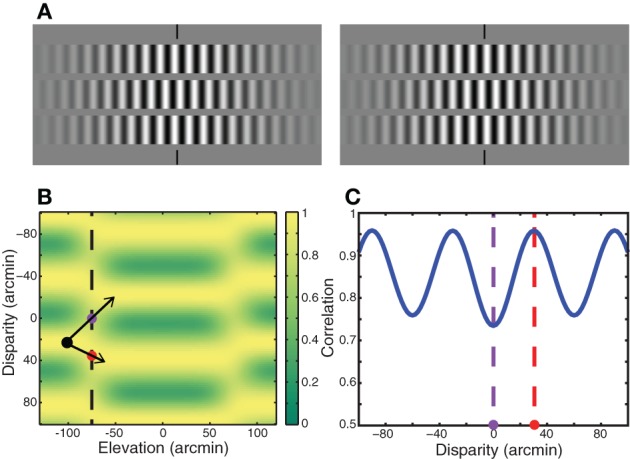
**Analysis of Zhang et al. (2001). (A)** The stereogram in the Zhang et al. study. A central strip composed of a sinewave grating is surrounded by flanking strips with gratings of the same frequency. Absolute-disparity minimization would lead to a percept in which the central strip is in front of the plane of fixation and the flanking strips are behind. Relative-disparity minimization would produce a percept in which the central and flanking strips are both in front of or both behind the fixation plane. Cross-fuse to see the stereoscopic percept derived from this stimulus. Most viewers perceive both the central and flanking strips in front of the fixation plane, the percept predicted by relative-disparity minimization. **(B)** Output of local cross-correlation conducted on this stimulus. The abscissa represents vertical position in the stimulus. The ordinate represents horizontal disparity; positive disparities are near and negative are far. The black dot represents one of the solutions for the flanking strip. The red dot is positioned in the transition between the flanking strip and a solution for the central strip that would minimize relative disparity. The purple dot is positioned in the transition between the flanking strip and a solution for the central strip that would minimize absolute disparity. **(C)** Correlation as a function of disparity along the vertical dashed line in panel **(B)**. Correlation ranges from 0.5 to 1. The purple dashed line represents the solution toward absolute-disparity minimization. The red dashed line represents the solution toward relative-disparity minimization. The correlation is higher in the region indicated by the red dot than in the region indicated by the purple dot. Thus, cross-correlation favors relative-disparity minimization rather than absolute-disparity minimization.

Zhang et al. (2001) devised stereograms in which matches that minimize absolute disparity were not the same as matches that minimize relative disparity. Their stimulus (Figure 8A) consisted of three horizontal strips: a central strip presented at one disparity and two flankers presented at another disparity. The strips were Gabor functions (i.e., sinewave carrier and Gaussian envelope) that yielded matching ambiguities similar to the stimulus in the upper half of Figure 1. When the central strip was viewed by itself, Zhang et al. found that the perceived solution minimized absolute disparity, which is consistent with the zero-disparity rule. However, when the flankers were present, they often observed a solution that minimized relative disparity instead of absolute disparity, which is consistent with the minimum relative disparity rule. Zhang et al. concluded that minimizing relative disparity often takes precedence over minimizing absolute disparity.

Many stereo phenomena including relatively low stereoresolution and the spatial disparity-gradient limit can be understood as a byproduct of estimating disparity by correlating the two eyes' images (Banks et al., 2004; Filippini and Banks, 2009; Allenmark and Read, 2011). For example, the disparity-gradient limit occurs because correlation tends toward zero when the two eyes' images differ substantially, as they must when the disparity gradient is high; when the correlation is low, correct matches are difficult to distinguish from false ones. The Zhang et al. stimulus creates large disparity gradients from the edge of a flanking strip to the adjacent edge of the central strip, so we wondered whether correlations in the transitions from flanker to center might favor the relative-disparity solution they frequently observed. We applied local cross-correlation to the Zhang et al. stimulus using parameters from previous analyses (Banks et al., 2004; Filippini and Banks, 2009). Figure 8B shows the output of local cross-correlation conducted on their stimulus. Correlation is plotted as a function of vertical position and disparity. Because the stimulus is periodic and therefore ambiguous, correlation varies as a periodic function of disparity. The region of interest is the transition from the central to flanking strips, which is indicated by the vertical dashed line in Figure 8B. The black dot is centered in the flanking strip closest to zero disparity. The minimum relative-disparity solution is indicated by the arrow down and to the right from the black dot through the red dot. The minimum absolute-disparity solution is indicated by the arrow up and to the right from the black through the purple dot. Figure 8C plots correlation along the vertical dashed line in Figure 8B. The correlation is higher in the transition zone for the relative-disparity solution (red dot) than it is in the transition zone for the absolute-disparity solution (purple dot). The higher correlation may bias the perceived solution toward relative-disparity minimization. We hasten to add, however, that the correlation output alone cannot dictate the final percept; processes beyond this stage of disparity estimation must be involved to prune the multitude of possible solutions. Our point is that correlation is higher in the transition from one strip to another that favors the smallest relative disparity than it is in the transition that favors the smallest absolute disparity.

### Does eccentric gaze trigger internal adjustments in image size?

When the eyes are in eccentric gaze, the pattern of horizontal disparities associated with a gaze-normal plane is altered because one eye is nearer to that plane than the other (Backus et al., 1999). Ogle (1940) and Bishop (1994, 1996) offered an intriguing hypothesis about how the visual system takes eccentric viewing into account when interpreting disparity at the retinas. They proposed that a neural mechanism, triggered by rotation of the eyes into eccentric gaze, causes an internal, overall magnification of one eye's image relative to the other. Thus, “a change in the relative functional sizes of the ocular images of the two eyes occurs in the vertical meridian when the eyes are turned in asymmetric convergence and in general this change is of an amount which offsets the difference in the distance of the observed object from the two eyes” (Ogle, 1940, p. 1038). Ogle and Bishop were suggesting therefore that the retinal images in the two eyes are internally adjusted in overall size according to sensed eye position (in this case, horizontal version). For leftward gaze, the right-eye's image would be internally magnified to offset the magnification that occurred due to the left eye being closer to the object. If the hypothesized internal adjustment of image size occurred before stereo correspondence, one would expect the interocular-frequency ratio at which Venetian blinds are observed to depend on horizontal version. Our data show that the critical ratio is mostly independent of eye position and are therefore inconsistent with Ogle's and Bishop's hypothesis.

The perception of size is also inconsistent with Ogle's and Bishop's hypothesis. Ogle (1939) used a stereoscope to present images of identical size to the two eyes and viewed those images with the eyes in eccentric gaze. Even though the retinal images were the same size, he reported that the image in the contralateral eye (i.e., the right eye in leftward gaze) appeared larger than the image in the ipsilateral eye. But this effect was almost certainly due to the asymmetric eye movements that occur in eccentric gaze (Schor et al., 1994) because the apparent size difference disappeared when Ogle held the eyes stationary. You can verify that no internal image-size adjustment occurs in eccentric gaze with a simple experiment. Hold a coin up close and view it in eccentric gaze. Wink your eyes back and forth and notice that the image appears larger in the ipsilateral eye than in the contralateral eye. You can also observe this keeping both eyes open by crossing the eyes in order to see double images. This difference in apparent size would not occur if eye position were used to internally adjust image sizes.

Finally, it is well known that stereo performance diminishes when images of different sizes are presented to the two eyes (Highman, 1977). Vlaskamp et al. (2009) showed that the inter-ocular size ratio at which stereo performance begins to decline does not depend on the direction of gaze. Thus, the influence of relative image size on stereo performance is also inconsistent with Ogle's and Bishop's hypothesis.

## Conclusion

When the binocular input is periodic—e.g., vertical sinewave gratings of potentially different spatial frequencies in the two eyes—the initial stage of disparity estimation yields multiple interpretations stacked in depth. But the percept has only one apparent depth in each visual direction. Thus, the multitude of possibilities is pruned down to one stable percept of a single depth in each direction. We showed that this pruning process includes a bias toward solutions that minimize absolute disparity—i.e., solutions that yield a surface near the horopter—rather than a bias toward solutions that minimize slant.

## Conflict of interest statement

The authors declare that the research was conducted in the absence of any commercial or financial relationships that could be construed as a potential conflict of interest.

## Supplementary material

Supplementary Video 1 **Demonstration of Venetian-blind effect as a function of frequency ratio**. Top: Two spatial frequency gratings are initially presented with a frequency ratio of one. The spatial frequencies of the gratings change in opposite sign until the frequency ratio is equal to 0.67. The frequencies of the gratings then converge until the ratio returns to one. Bottom: The cross correlation output is shown for each frame of the video. Green regions represent high correlation while black regions represent low correlation. Azimuth is shown in the abscissa and corresponds to azimuth in the gratings above; high correlation is computed for all disparities beyond the edge of the gratings because there are no unique features to constrain matching. The ordinate of the correlation output represents disparity and changes from crossed disparity (in front of the screen) at the bottom of the correlation output to uncrossed disparity (behind the screen) at the top of the correlation output; zero disparity is denoted by the white line.
